# Synthesis and Characterization of Novel Hybrid Wollastonite–Metakaolin-Based Geopolymers

**DOI:** 10.3390/ma17174338

**Published:** 2024-09-02

**Authors:** Mazen Alshaaer, Abdulaziz O. S. Alanazi

**Affiliations:** 1 Department of Physics, College of Science and Humanities in Al-Kharj, Prince Sattam bin Abdulaziz University, Al-Kharj 11942, Saudi Arabia; abdulazizaoda@gmail.com; 2 Department Mechanics of Materials and Constructions, Vrije Universiteit Brussels (VUB), Pleinlaan 2, 1050 Brussels, Belgium

**Keywords:** calcium silicates, functional geopolymers, XRD, Ca_5_(SiO_4_)_2_(OH)_2_, calciochondrodite

## Abstract

Over the past few decades, researchers have focused on developing new production methods for geopolymers to improve their properties for use in multiple applications as a functional material. This study introduces a new geopolymer system based on wollastonite and metakaolin as precursors. The role of wollastonite was also explored alongside metakaolin in geopolymers. Geopolymers were synthesized by adding wollastonite to metakaolin in different ratios: 0 wt.%, 12.5 wt.%, 25 wt.%, and 50 wt.%. The alkaline activator was then mixed with the powder, wollastonite, and metakaolin to prepare the geopolymers. In addition to mechanical tests, the hardened geopolymers were characterized using XRD, TGA, and SEM techniques. The findings revealed that adding wollastonite in amounts of 0 wt.%–12.5 wt.% did not affect the strength of the geopolymers. Increasing wollastonite between 25 wt.% and 50 wt.% significantly increased the geopolymers’ flexural and compressive strength from 3 MPa to 12.3 MPa and from 23 MPa to 54 MPa, respectively. The use of wollastonite as a precursor also led to fundamental changes in the microstructural structure of the geopolymer matrix: a new crystal phase, (Ca_5_(SiO_4_)_2_(OH)_2_), calciochondrodite was formed, and the Si-Al-Na crystal phase disappeared, leading to significant changes in the amorphous phase.

## 1. Introduction

The process of geopolymer cement [1] begins with the alkali activation of aluminum silicates, which form stable, hard materials of a tectosilicate-like structure [2,3,4,5]. Geopolymers are attractive because they harden at low temperatures, demonstrate excellent mechanical performance, and are functional materials for various applications. Their use is possible in construction, waste recycling [6], water treatment, toxicity stabilization, and passive cooling [7]. Unfortunately, using large amounts of alkaline solutions in field applications can be technically difficult. Furthermore, the higher cost of geopolymers than ordinary cement makes large-scale use of this material challenging. To address these challenges, researchers are developing strategies to market geopolymers as “green” and environmentally friendly materials with multiple uses in engineering and environmental applications [8,9].

The calcination of kaolinite at an appropriate temperature produces metakaolin (MK) [10]. Metakaolin is a structurally amorphous product produced by dehydrating kaolin, namely its essential component kaolinite. Due to significant improvements in the mechanical characteristics of geopolymers, special attention was paid to adding other precursors and fillers to the system, thereby maintaining good performance and minimizing production costs [11]. One functional geopolymer is based on the synthesis of mesoporous and macroporous geopolymer-based materials. These materials have the dual function of improving thermal insulation and reducing noise pollution [12]. Negative environmental factors during the processing and cost impacts are minimized when untreated kaolin is used instead of metakaolin. Zeolite belongs to aluminosilicate-based minerals or tectosilicates, which contain alkali earth metals or alkali metals. These minerals exhibit high ion exchange, adsorbability, heat resistance, acid resistance, and catalysis [11]. The geopolymer synthesized by alkali activation of zeolitic tuff and natural zeolite exhibits excellent mechanical characteristics and has a similar porous structure to zeolite [13]. Aluminum, iron, and aluminosilicates are the main ingredients in laterite minerals. The traditional use of laterite as a building material, road, and brick is based on its strong corrosion resistance, which is why it is mostly reddish-brown. Lateritic, which has high mechanical performance, has become a common material for geopolymer production. Furthermore, laterite’s chemical composition has a suitable molar oxidation rate compared to other minerals, which is used as a precursor for the Na-poly (sialate–siloxo) geopolymer. The microstructure, phase composition, and mechanical properties and of the laterite-based geopolymer are greatly impacted by the molar oxide ratio of silica to alumina [14]. Furthermore, blending laterite with other solid wastes produces high-strength geopolymers. Non-load-bearing construction materials [15] can benefit from the good application prospects of laterite and mixed laterite–slag geopolymers. Diatomite [16], mullite, bauxite [17], bentonite [18], mullite [19], and halloysite [20] are commonly known natural minerals that can be used as precursors of geopolymers and are aluminosilicate materials [11]. Thermal treatment, which aims to increase the amorphous characteristics of some natural minerals, may be necessary to increase their reactivity during geopolymerization reactions. Future research must develop geopolymer materials with low energy consumption. By adding sulfur, which is a waste byproduct, to geopolymer precursors, the mechanical performance of MK-geopolymers was found to improve [15].

Enhancing the mechanical performance and elastic properties of end geopolymer products requires fillers as well as synthetic and natural reinforcement materials. The geopolymeric matrix can be strengthened by fibers fabricated from minerals [21], glass fibers [22], polymers [23,24], metals [25], and carbon. Geopolymer composites improve their mechanical performance by enhancing their ductility, tensile strength, and toughness. Natural fibers, waste, and recycled minerals are preferable as reinforcements because they are considered environmental factors. For instance, the potential use of luffa cylindrical fibers in metakaolin-based geopolymer composites as a reinforcement has been explored [7] with promising results: flexural strength increased from 3 MPa to 14 MPa and compressive strength increased from 13 MPa up to 31 MPa [26]. Fly ash is a readily available byproduct of geopolymer preparation, which is a common use of raw materials [27]. Future research must develop geopolymer materials while consuming less energy.

Calcium silicate, also known as wollastonite, is known for its natural formation of aggregates of needle-shaped or acicular crystals. This mineral is characterized by its low cost, high whiteness, low thermal expansion coefficient, chemical stability, and hardness [28]. To produce composites with high stiffness and toughness [28,29], very fine wollastonite is applied as an inorganic filler in elastomers and plastic-based blends. A new cement, vubonite, is prepared by mixing wollastonite with phosphoric acid and heavy metal oxides to meet high-temperature and construction applications [30]. The use of wollastonite as a functional filler in thermoplastic composites could significantly reduce the cost of these composites, which is less expensive than fiberglass [31]. The mechanical characteristics of oil well cement can be enhanced by combining wollastonite fiber with carbon fiber [32]. 

Previous studies [33] showed that flexural strength was not improved by wollastonite as a geopolymer precursor and compressive strength increased slightly or remained unchanged. Therefore, the novelty of this work is to explore the possibility of synthesizing a new hybrid geopolymer using combinations of metakaolin and wollastonite as precursors. The chemical, microstructural, and mechanical properties of wollastonite–metakaolin-based geopolymers will be discussed and analyzed in this paper. 

## 2. Materials and Methods

### 2.1. Materials 

The process of synthesizing geopolymer cement involved the use of kaolinitic clay, Na_2_SiO_3_ solution, and NaOH solution. The untreated kaolinitic soil sample [26] was collected from a kaolinite deposit in the Riyadh region (Saudi Arabia) with the help of the Saudi Ceramic Company. Table 1 provides information about the chemical composition of calcined kaolinitic soil (metakaolin). Based on the loss of ignition oxides Si and Al, the precursor was estimated to contain around 90% of the kaolinite mineral [26]. The amorphous kaolinite (metakaolin) was obtained by heating the kaolinitic soil in a furnace at 750 °C for 4 h (Nabertherm, New Castle, DE, USA). Wollastonite (CaSiO_3_) [34] with fibrous crystals (NYAD^®^ 200, NYCO, Paris, France) was used as a secondary precursor.

The alkaline activator was prepared using Na_2_SiO_3_, NaOH, and H_2_O. The solution of sodium hydroxide (NaOH) was prepared using pure pellets of sodium hydroxide (Merck, Darmstadt, Germany) and deionized water. The Na_2_SiO_3_ solution consisted of 27 wt.% SiO_2_ and 8 wt.% Na_2_O [35,36].

### 2.2. Synthesis of the Geopolymer Samples

The solutions of sodium silicate, sodium hydroxide, and metakaolin powder of all series were prepared based on the following molar ratio of Si/Al/Na: 1.6/1/1, respectively. The resultant alkaline solution had a 6.3:1 molar ratio of H_2_O/Na_2_O. This solution was created by initially stirring NaOH, H_2_O, and Na_2_SiO_3_ for 6 h. The alkali solution was mixed using mechanical mixing after adding the metakaolin–wollastonite mixture. The content of the wollastonite was increased from 0% (GW0) to 50 (GW50) (compared with the weight of the metakaolin) and is the maximum limit of the Powder-to-Liquid ratio while maintaining reasonable workability. The use of wollastonite as a replacement for metakaolin was examined in previous studies [33] and did not show an improvement in the mechanical properties of the geopolymer.

**Figure 1:**
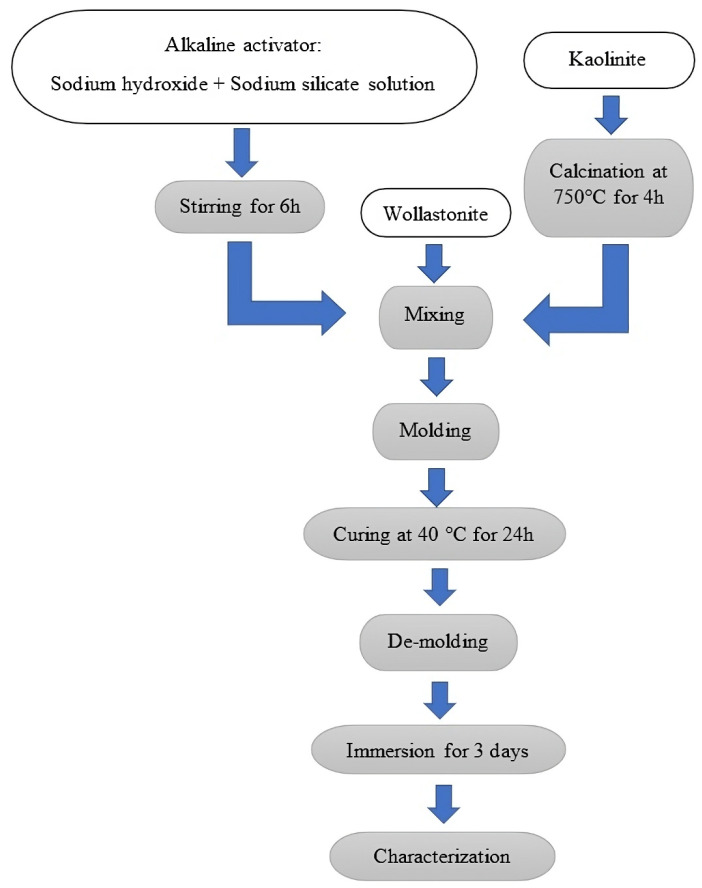
Experimental procedure.

The geopolymer mixture was finally poured into silicon molds measuring 20 mm × 20 mm × 120 mm, sealed, and placed in an oven (Raypa Company, Barcelona, Spain) at 40 °C for 24 h for curing. To assess their stability in the presence of water, the hardened geopolymers were demolded and placed in water for 3 days. The specimens were then subjected to various characterization methods. Table 2 and Figure 1 display the geopolymer series compositions and preparations. The alkaline solution and metakaolin are the sources of SiO_2_. Alkali activator solutions supplied sodium oxide (Na_2_O) while metakaolin provided aluminum oxide (Al_2_O_3_). Wollastonite, as the proposed precursor in this study, could contain both CaO and SiO_2_.

### 2.3. Characterization Techniques

The morphology and microstructure of each series of the geopolymers were examined by coating the samples with platinum and scanning them with a SEM (Quanta Inspect F50, FEI Company, Eindhoven, The Netherlands). TGA (TG 209 F1 Libra, Netzsch, Germany) was used to measure the thermal properties and mass loss with heating when heating the samples (minimum 100 mg). Temperatures ranged from 50 °C to 800 °C at a rate of 2 °C per minute. A helium-filled environment was used to conduct this test. The samples underwent qualitative mineralogical and phase analysis using a Shimadzu XRD diffractometer-6000 (Nishinokyo Kuwabara-cho, Nakagyo-ku, Kyoto, Japan) with a cobalt tube and a 2-theta scanning range of 5–80° at a 2°/min scan rate. The software MATCH! was used to perform a Rietveld refinement of the materials produced (Version 4, Crystal Impact, Bonn, Germany).

Mechanical testing was conducted on the specimens using three-point bending and compression. A universal testing machine (HD-B615-S, China) was used to test at room temperature. This testing was performed on three specimens from each series. The measurements of the bending specimen were 20 mm, 20 mm, and 120 mm. The distance between the supports was 80 mm, and the machine head could move at 0.1 mm/minute during testing. The failed bending specimens underwent compression tests, which involved placing them on their sides with a loading area of 20 × 20 mm^2^ and a height of 40 mm. The machine head was running at a speed of 2 mm per minute while being tested. The following equation was used to determine flexural strength (Sr):

$$S_{r}=\frac{3}{2}\frac{F_{m}S}{BW^{2}}$$

where *F_m_* is the maximum load (N), *S* is the span of the sample, *B* is the specimen width, and *W* is the specimen thickness (depth).

The compressive strength (*S_c_*) was calculated using the following formula:

$$S_{c}=\frac{F_{C}}{A}$$

where *F_c_* is the maximum load (N), and *A* is the cross-section area of the specimen (mm^2^).

To examine the microstructure and morphology of the geopolymers, the sample was coated with platinum and scanned using a scanning electron microscope (SEM) (Quanta Inspect F50, FEI Company, Eindhoven, The Netherlands). The samples (~100 mg) were heated using TGA (TG 209 F1 Libra, Netzsch, Germany) to measure the thermal properties and mass loss with heating. The temperature range ranged from 50 °C to 800 °C and had a rate of 2 °C/min increments. A helium-filled environment was used for this test. The sample was analyzed qualitatively in terms of mineralogical and phase parameters using a Shimadzu XRD diffractometer-6000 (Japan) with a cobalt tube and a scanning range of 5°–80° at a 2°/min scan rate. The resultant XRD data underwent a Rietveld refinement in MATCH! software (Version 4, Crystal Impact, Bonn, Germany) using the fundamental parameter approach.

## 3. Results and Discussion

### 3.1. Effect of Wollastonite Loading on Mechanical Performance

**Figure 2:**
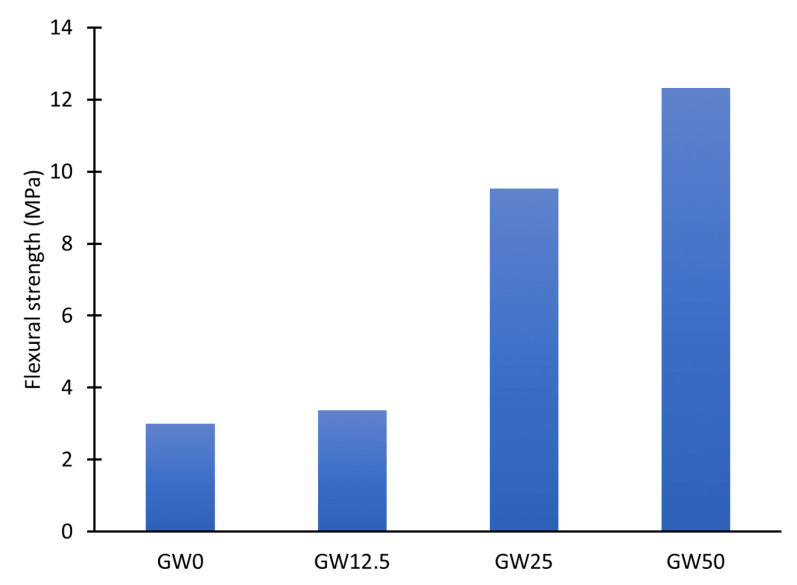
Flexural strength as a function of wollastonite weight percentage.

The mechanical characterization shows that wollastonite significantly affects the geopolymer’s mechanical performance based on its function as a precursor and filler. Figure 2 illustrates variations in the compressive strength of the specimens (Table 2). A slight increase in the geopolymer’s flexural strength was observed by increasing the load of wollastonite from 0 wt.% to 12.5 wt.%, as demonstrated by the comparison with metakaolin. The flexural strength increased from 3 MPa to 9.5 MPa when wollastonite was loaded with 25 wt.% of the geopolymer. Then, the wollastonite weight percentage was increased to the highest level to maintain the workability of the geopolymer mix. Meanwhile, the flexural strength increased fourfold after incorporating wollastonite into the matrix. The increase to the upper limit of wollastonite loading, 50 wt.%, resulted in a fourfold increase in flexural strength from 3 MPA to 12.3 MPA. 

**Figure 3:**
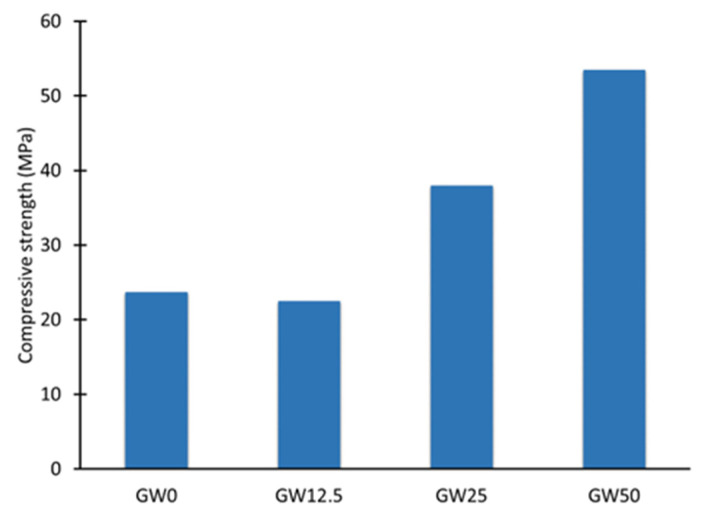
Compressive strength as a function of wollastonite weight percentage.

Figure 3 shows that introducing wollastonite to the geopolymer matrix causes a sharp increment in compressive strength. The geopolymer exhibits a compressive strength behavior similar to the flexural strength, as shown in Figure 3, respectively. The geopolymer’s compressive strength increases from 23 MPa (GW0) to 40 MPa (GW25) after adding a wollastonite percentage of 25. Increasing wollastonite up to 50% (GW50) increases compressive strength to 54 MPa (Figure 3). According to the mechanical properties of the geopolymers, the optimal wollastonite contents of WK-geopolymer vary from 25% (GW25) to 50% (GW50). 

These results confirm that a minimum percentage of wollastonite should be used to demonstrate its effect on geopolymers’ mechanical properties. Using wollastonite with lower ratios may not improve mechanical properties, which explains the results of previous studies [33] where wollastonite did affect geopolymers’ mechanical performance. 

### 3.2. Analysis of Microstructure and Phase Composition

**Figure 4:**
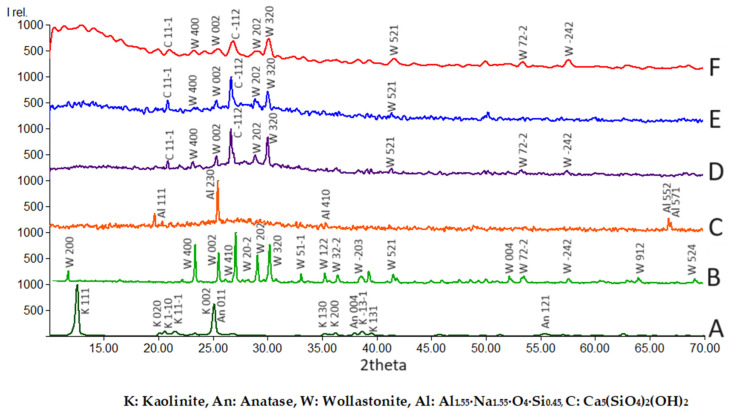
Qualitative XRD patterns for (A) Kaolinite, K; (B) wollastonite, W; (C) MK-geopolymer, GW0; (D) GW12.5; (E) GW25; and (F) GW50.

To determine the role of wollastonite in the matrix of geopolymers, an XRD analysis was carried out. Figure 4A shows XRD scan analyses of kaolinite (K); wollastonite (W); the metakaolin-based geopolymer (GW0); and the WK–geopolymers (GW12.5, GW25, and GW50). The characteristic XRD peaks of the kaolinite and wollastonite samples are presented in Figure 4A,B, respectively. The XRD pattern in Figure 4A shows that anatase minerals (TiO_2_) with Miller indices of (011) and (121) are present in kaolinite in an amount of 10.8% [26] (Table 3). The standard unit cell volume of kaolinite is 320.1 Å^3^, while the unit cell volume here is 329.4 Å^3^, which differs slightly from typical kaolinite. The presence of impurities such as iron oxides could change the unit cell volume of kaolinite [36]. Typically, kaolin clay coexists with anatase [37], which is a metastable mineral form of titanium dioxide (TiO_2_) with a tetragonal crystal structure [38].

The XRD pattern shows several peaks for wollastonite (Figure 4B) at 2θ = 11.74, 23.38, 27.08, and 30.20. Wollastonite (W) is characterized by a monoclinic crystal structure with a unit cell specification of a = 15.42 Å, b = 7.32 Å, c = 7.07 Å, and β = 95.37° (Table 3).

Due to the calcination of kaolinite and the subsequent geopolymerization in an alkaline environment, the XRD peaks of the kaolinite and anatase minerals disappeared, as reported in Figure 4C for GW0 (MK-geopolymers). As shown in Figure 4C, a high background for the XRD patterns, between 15° and 35°, confirms the presence of an amorphous phase in the produced MK-geopolymer [26,36]. According to the Rietveld refinement analysis of the XRD patterns, the degree of GW0 crystallinity was 42%. The detected crystalline phase of GW0 was orthorhombic Al_1.55_·Na_1.55_·O_4_·Si_0.45_, (Table 4). As shown in Figure 4D–F, introducing wollastonite to the MK-geopolymer diminished all the peaks corresponding to Al_1.55_·Na_1.55_·O_4_·Si_0.45_ and formed new crystalline phases (Ca_5_(SiO_4_)_2_(OH)_2_) and calciochondrodite minerals besides several peaks corresponding to wollastonite. The structure of calciochondrodite is very similar to that of Reinhardbraunsite Ca_5_(SiO_4_)_2_(OH,F)_2_. The XRD pattern shows peaks for calciochondrodite in Figure 4D–F, at 2θ = 20.87, 26.65, 30.82, and 30.99, respectively. Calciochondrodite (C) is characterized by a monoclinic crystal structure with a unit cell specification of a = 11.42 Å, b = 5.05 Å, and c = 8.93 Å, β = 109.3°. The major crystallographic planes corresponding to the Miller indices of (11$\overset{-}{1}$) and ($\overset{-}{1}$12) were observed to display crystal expansion.

**Figure 5:**
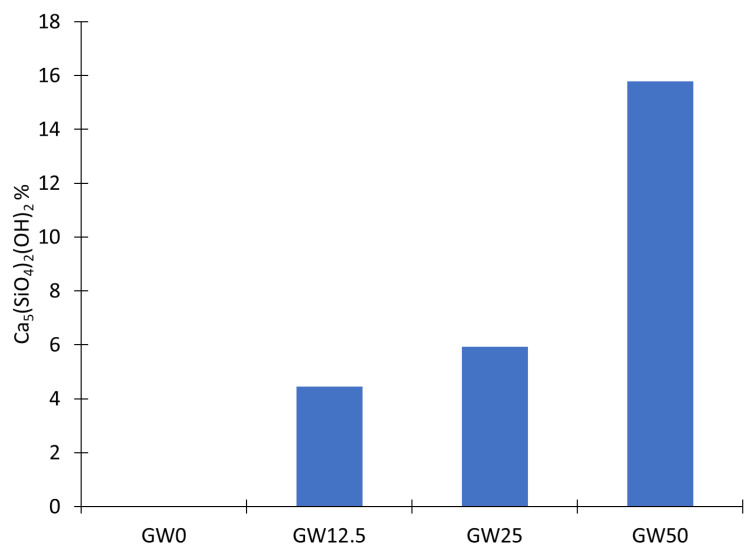
Reinhardbraunsite (Ca_5_(SiO_4_)_2_(OH)_2_) contents as a function of loading wollastonite into the geopolymer.

According to the Rietveld refinement analysis of the XRD data of GW12.5, GW25, and GW50, two crystalline monoclinic phases are detected: a new phase of Ca_5_(SiO_4_)_2_(OH)_2_, and wollastonite with different weight contents (Table 4). These results confirm the incorporation of wollastonite as a reactive filler and precursor in the geopolymer matrix alongside metakaolin. Initially, the degree of crystallinity decreased sharply from 42% to 12% with the addition of wollastonite, GW12.5, to the metakaolin as precursors. Adding more wollastonite, GW25, and GW50 increases crystallinity by 20% and 49.5%, respectively. Figure 5 illustrates an increasing trend in Ca_5_(SiO_4_)_2_(OH)_2_ contents as a function of loaded wollastonite. This result confirms that Ca_5_(SiO_4_)_2_(OH)_2_ is formed by wollastonite hydration during geopolymerization. 

The XRD hump corresponding to the amorphous phase was detected between 22 °C and 35 °C. The crystalline phases of GW25 differed from those of the metakaolin–polymer, GW0, where a new phase associated with wollastonite appeared, known as Ca_5_(SiO_4_)_2_(OH)_2_ (Table 4). The main crystalline phase, Al1_.55_·Na_1.55_·O_4_·Si_0.45_, of the MK-geopolymer, GW0, disappeared with loading wollastonite. There was also a decrement in wollastonite crystalline sizes after polymerization from 4479 nm (Table 3) to 3264 nm (Table 4), indicating wollastonite’s partial reactivity as a filler. 

There is a clear correlation between Ca_5_(SiO_4_)_2_(OH)_2_ contents (Figure 5) as a geopolymer phase and improving the mechanical properties of geopolymers (Figure 2 and Figure 3). The results showed that the flexural and compressive strength of the geopolymer improved significantly when wollastonite was used as a mineral filler and precursor. The reaction mechanism of wollastonite differed from that of a metakaolin-based geopolymer. The metakaolin significantly promoted the polymerization reaction of Si–Al gels and wollastonite could activate C–S–H gels as a source of calcium and silica. The Si–Al and C–S–H gels strengthened the geopolymer system [39] when wollastonite contents were above 25% of metakaolin, GW25, and GW50, Figure 2 and Figure 3. However, C–S–H gels and Ca_5_(SiO_4_)_2_(OH)_2_, which are formed by the hydration of wollastonite, strengthened the geopolymer. In WK-geopolymers with a wollastonite/metakaolin ratio above 25% (GW25 and GW12.5), the hydration reaction of wollastonite and the polymerization reaction of the Si–Al gels promoted each other.

**Figure 6:**
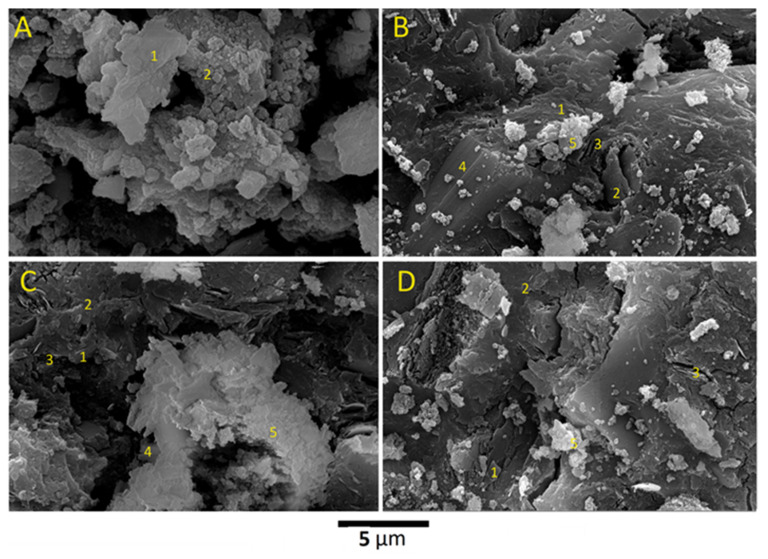
SEM images of (**A**) GW0, (**B**) GW12.5, (**C**) GW25, and (**D**) GW50; (1) metakaolin layers, (2) MK-geopolymer matrix, (3) voids resulting from the dissolution of metakaolin, (4) wollastonite, and (5) WK-geopolymer matrix.

Figure 6A (point 1) depicts residual metakaolin layers as the primary precursor. Sources of aluminum ions for the MK-geopolymer, GW0, are illustrated by the SEM. The SEM analysis shown in Figure 6A (point 2) shows that the microstructure of GW0 is characterized by binder material (Na–Al–Si-based geopolymer matrix) and partially dissolved metakaolin layers (point 1). This SEM image also shows that, as a result of geopolymerization and the setting reactions, the voids between partially dissolved metakaolinite layers were filled with sodium aluminosilicate matrix [26]. The produced WK-geopolymers, GW12.5, GW25, and GW50, exhibited a different microstructure, as reported in Figure 6B, Figure 6C, and Figure 6D, respectively. Wollastonite crystals, which are strongly associated with the geopolymer matrix, can be observed in Figure 6B (point 4). In addition, random monoclinic crystals of Ca_5_(SiO_4_)_2_(OH)_2_ are associated with the geopolymer matrix, as reported in Figure 6B–D (point 5). 

**Figure 7:**
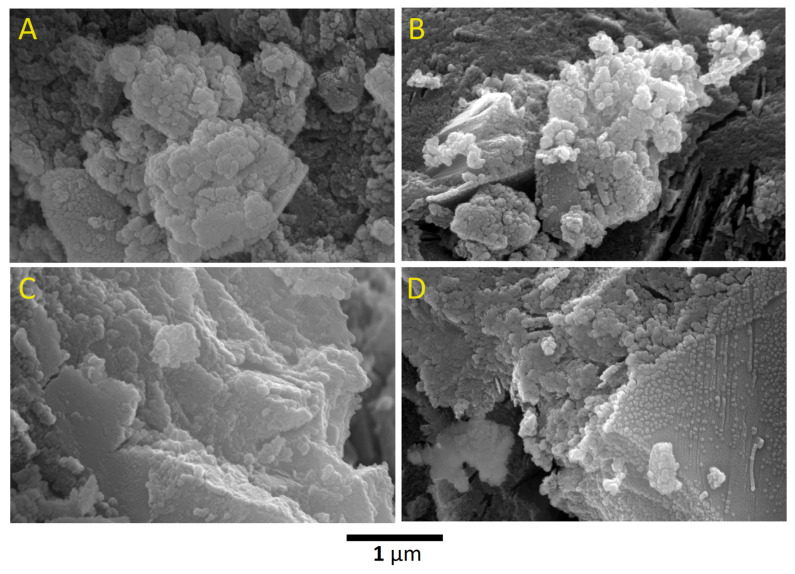
SEM images with a high magnification of (**A**) GW0, (**B**) GW12.5, (**C**) GW25, and (**D**) GW50.

The nano-pattern observed in the microstructures of GW0, GW12.5, GW25, and GW50 can be attributed to the encapsulation of nanoparticles in the geopolymer matrix (Figure 7A), GW0. These results are consistent with previous studies, where amorphous or microcrystalline sodium aluminosilicates were formed as a result of geopolymerization [5]. Clusters of nanoparticles are present in the microstructure of WK-geopolymers, GW12.5, GW25, and GW50 in Figure 7B, Figure 7C, and Figure 7D, respectively, after adding wollastonite.

### 3.3. Thermogravimetric Analysis (TGA) 

**Figure 8:**
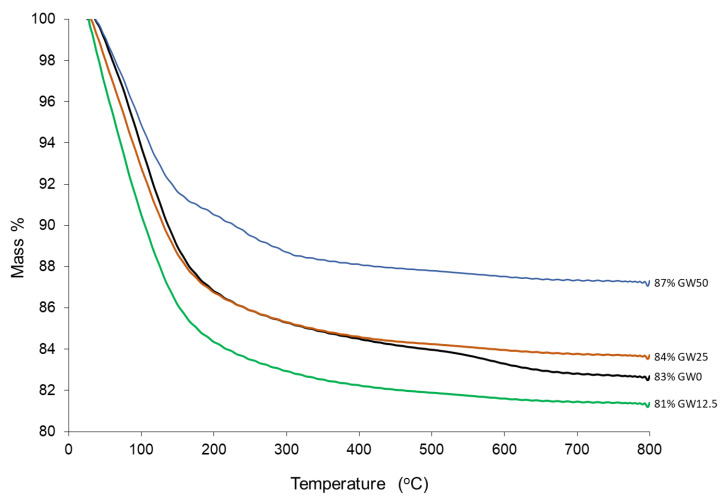
TGA of geopolymers.

The thermal decomposition process of the produced geopolymers, GW0, GW12.5, GW25, and GW50 in air was investigated using TG tests. The corresponding results are shown in Figure 8. Total weight loss as a result of heating the GW0 (MK-geopolymer) to 800 °C was around 17%. After adding wollastonite, the cumulative mass loss of WK-geopolymers varied from 19% (GW12.5) to 13% (GW50). The changes in mass loss were not systematic compared to GW0 with the addition of wollastonite, particularly GW12.5. The major factors of mass loss were weakly bonded water and structural water (Table 4).

**Figure 9:**
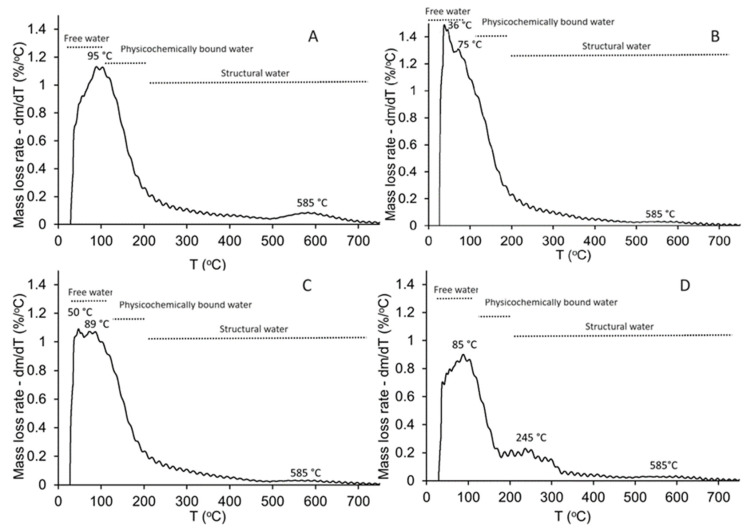
Differential TGA of the geopolymers; (**A**) GW0, (**B**) GE12.5, (**C**) GW25, and (**D**) GW50.

According to Figure 9A, thermal events occur in two main temperature ranges, 50–250 °C and 500–700 °C. Free water and fine-pore moisture release is associated with weight reduction in the first range (25–200 °C). Zeolitic water release from the nanoporous network can cause a decrease in weight in the second range (500–700 °C) [13]. Adding 12.5% of wollastonite to the geopolymer precursors, GW12.5, increases the overall mass loss, particularly at temperatures below 100 °C (Figure 9B), or free water. The mass loss attributed to zeolitic water at 585 °C decreases compared to GW0, indicating that using a small percentage of wollastonite could negatively affect geopolymerization by producing smaller ratios of geopolymer binder material. These results are supported by the limited effect on mechanical properties, as reported in Figure 2 and Figure 3. Increasing the wollastonite to 25% (GW25) shifts the evaporation temperature, physicochemically bound water, and structural water toward higher temperatures (Figure 9C). The presence of high wollastonite contents in the microstructure of geopolymers, GW50, leads to mass loss peaks between 200 °C and 300 °C, with the next step observed at 585 °C. The decarbonation of wollastonite at different temperature ranges is one of the factors attributed to weight loss in these two steps [40].

### 3.4. The Physical Role of Wollastonite in the Hybrid WK-Geopolymer

**Figure 10:**
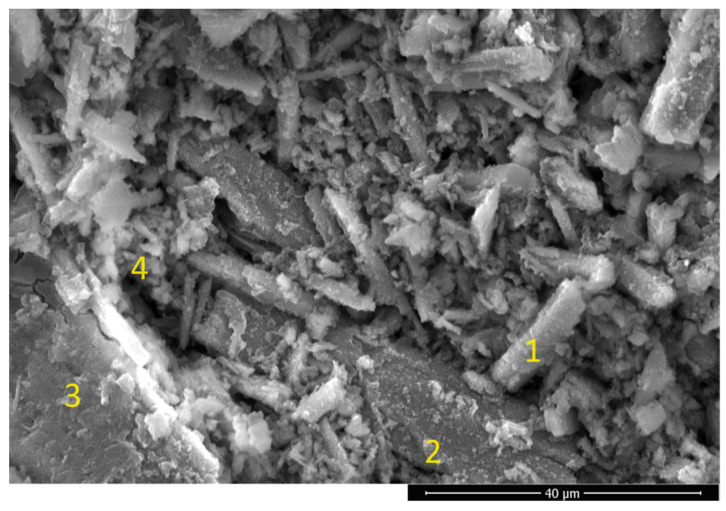
Powdered WK-geopolymer (GW25); (1) wollastonite, (2) metakaolin, (3) WK-geopolymer matrix, (4) calciochondrodite.

The above-mentioned results show that wollastonite has a major chemical effect on the microstructure of geopolymers, whether by forming new crystalline phases or by changing the amorphous phase. To study the physical effects of wollastonite on geopolymers, a sample of GW25 was powdered and examined using a SEM. Figure 10 depicts different matrix phases, especially residual or partially reactive wollastonite grains that are clearly visible (point 1). Random orientation of wollastonite microfibers with a 10 µm length and 1 µm diameter can be observed in the powdered geopolymer, GW25. These microfibers are well bonded to the bulk matrix, as reported in Figure 6B (point 4). These findings are consistent with previous observations in Table 3 and Table 4 of a reduction in the size of residual wollastonite grains after geopolymerization, indicating the reactivity of the surface of these granules. Thus, chemical bonds could be created on the surface and strongly incorporate the wollastonite into the geopolymer matrix. In other words, the remaining wollastonite microfibrous crystals play an active role in strengthening the geopolymer matrix and act as a reinforcement of the microfiber composite. 

The above results show that residual wollastonite is used to produce functional geopolymer microfiber-reinforced geopolymer composites with multiple uses, such as construction, water purification, or thermal insulation.

## 4. Conclusions

In this study, emphasis was placed on integrating wollastonite with metakaolin to prepare a novel type of geopolymer with attractive mechanical properties and promising applications. This study has shown that adding wollastonite as a precursor alongside metakaolin leads to a complete change in geopolymers’ microstructure, phase composition, and mechanical characteristics.

X-ray analyses have shown that crystalline Na-Si-Al phases, i.e., Al_1.55_·Na_1.55_·O_4_·Si_0.45_, completely disappear and a new crystalline phase, calciochondrodite Ca_5_(SiO_4_)_2_(OH_)2_, emerges. This phase may play an essential role in the emergence of strong C–S–H bonds. This study showed that adding a small amount of wollastonite, i.e., 12.5%, is enough to significantly change the microstructure of the geopolymers. As a result of incorporating wollastonite and metakaolin as precursors, the amorphous phase increases dramatically from 58% to 88% according to MATCH! software analysis. However, the proportion of the amorphous phase decreases to 50.5% as the wollastonite amounts increase to 50% of the metakaolin.

Although a small amount of wollastonite can cause significant changes in the microstructure and chemical phases, limited changes were observed in the mechanical properties of geopolymers. Above a specific proportion, wollastonite must be added to the precursors to obtain high-strength geopolymers. Increasing wollastonite contents to 25% and 50% resulted in microstructural changes along with significant improvements in mechanical performance, where flexural strength increased fourfold from 3 MPa to 12.3 MPa. Compressive strength also doubled from 23 MPa to 54 MPa. In addition to chemical and microstructural changes, residual micro-fibrous wollastonite effectively acts as a filler and microfiber reinforcement of the geopolymer matrix.

This preliminary study undoubtedly presents many opportunities and challenges for developing promising new hybrid geopolymers with attractive characteristics and mechanical properties.

## Figures and Tables

**Table 1:** Chemical composition of untreated kaolinite.

Oxide	wt.%
MnO	0.34
Cr_2_O_3_	0.45
CaO	1.11
K_2_O	0.12
P_2_O_5_	0.93
Fe_2_O_3_	9.37
Al_2_O_3_	22.56
SiO_2_	38.41
TiO_2_	14.22
Loss on Ignition	13.10

**Table 2:** Mixes and composition of the prepared geopolymers.

ID	(Weight Ratios per 100 g of Metakaolin)				
	Metakaolin	Wollastonite	Na_2_SiO_3_ Solution	NaOH	H_2_O
GW0	100	0	100	25	48
GW12.5	100	12.5	100	25	48
GW25	100	25	100	25	48
GW50	100	50	100	25	48

**Table 3:** Results of the precursors’ XRD analysis (Rietveld refinement, MATCH! software, version 4).

Precursor	Phase	Phase %	Crystal Structure	a (Å)	b (Å)	c (Å)	V (Å^3^)	Crystallite Size (Å) ***
Kaolinite	Al_2_Si_2_O_5_(OH)_4_	89.2	Triclinic *	5.15	8.94	7.40	329.4	519
	Anatase TiO_2_	10.8	Tetragonal	3.78	-	9.51	135.9	425
Wollastonite	CaSiO_3_	100	Monoclinic **	15.42	7.32	7.07	794.5	4479

* α = 91.7°, β = 104.7°, γ = 89.9°. ** β = 95.37°. *** Scherrer equation.

**Table 4:** Rietveld refinement of XRD data showing the phase composition, crystallinity, and crystal parameters of the geopolymers (MATCH! software, version 4).

Series	Degree of Crystallinity	Crystalline Phase Composition	Phase%	Crystal System	Unit cell size (Å^3^)	Crystalline Size (Å)
GW0	42%	Al_1.55_·Na_1.55_·O_4_·Si_0.45_	100	orthorhombic	765.6	2820
GW12.5	12%	Ca_5_(SiO_4_)_2_(OH)_2_	37.1	monoclinic	486.1	1081
		CaSiO_3_	62.9	monoclinic	794.3	3430
GW25	20%	Ca_5_(SiO_4_)_2_(OH)_2_	29.6	monoclinic	486.1	2691
		CaSiO_3_	70.4	monoclinic	794.3	3699
GW50	49.5%	Ca_5_(SiO_4_)_2_(OH)_2_	31.9	monoclinic	486.1	522
		CaSiO_3_	68.1	monoclinic	794.3	3264

## Data Availability

The data that support the findings of this study are available from the corresponding author, M. Alshaaer, upon reasonable request.
